# Supplementary material for: Electric-field-driven non-volatile multi-state switching of individual skyrmions in a multiferroic heterostructure
Source: Nat Commun. 2020 Jul 17;11:3577. doi: 10.1038/s41467-020-17354-7 (PMC7367868; doi:10.1038/s41467-020-17354-7)
Supplement: Supplementary file 1 — Supplementary Information [file 41467_2020_17354_MOESM1_ESM.pdf]

Supplementary information for

Electric-field-driven Non-volatile Multi-state Switching of Individual Skyrmions in a Multiferroic Heterostructure

Wang et al.

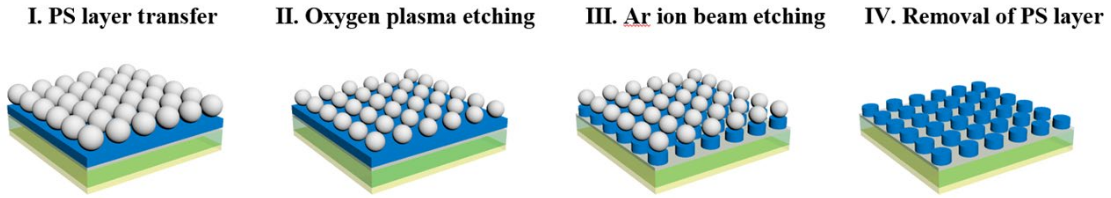

Supplementary Figure 1. Schematic diagrams showing the fabrication of the ferromagnetic ordered nano-dots by using a two-step nano-patterning method. (I) The monolayer polystyrene sphere (PS) particle arrays are transferred to the surface of magnetic film. (II) Subsequently, the PS particles are etched to an expected diameter by using oxygen plasma. (III) Next, the Ar ion beam is applied to etch the magnetic film with an appropriate etching time. During this process, the particles act as the etch mask. As a result, the regions of magnetic film blocked by PS particles are protected while the rest is etched. (IV) Finally, the PS particles are removed by chloroform solution and the periodically ordered magnetic nano-dot arrays are obtained.

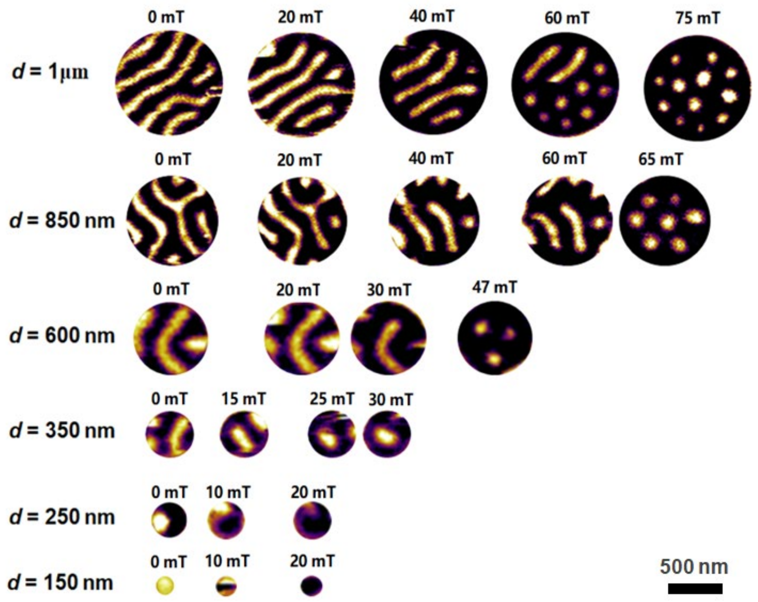

Supplementary Figure 2. The external magnetic field dependent domain evolution process in the $[Pt/Co/Ta]_{12}$ nano-dots with diameter (d) ranging from 1 μm to 150 nm. H represents the external magnetic field except that from the MFM tip. For the d ~ 1 μm nano-dots, the stripe domains are observed at $\mu_0 H$ = 0 mT. At a critical magnetic field ($\mu_0 H_c$) of 75 mT the stripe domains completely transform into skyrmions and the maximum number (N) of skyrmions that the nano-dot can host is 12. With the decrease of d, both $\mu_0 H_c$ and N decrease correspondingly. When d is equal to 350 nm, a single skyrmion could stabilize in the nano-dot at $\mu_0 H_c$ = 25 mT. By further decreasing d, the skyrmion cannot form anymore; however, the single domain starts to appear at d ~ 150 nm. The scale bar is 500 nm.

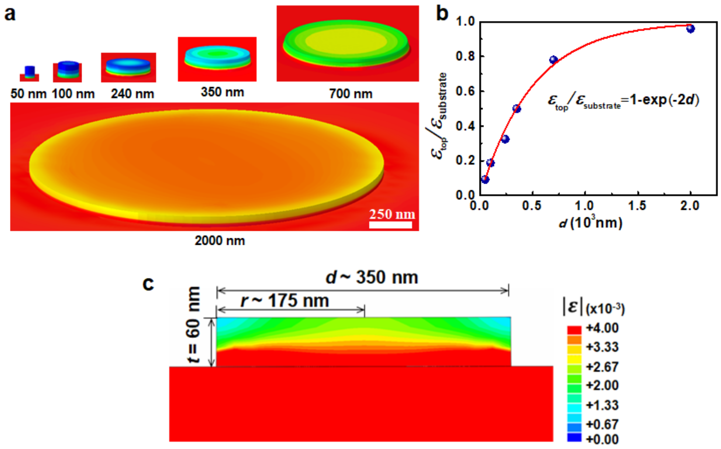

Supplementary Figure 3. **a** Finite element contour plots of strain on nano-dots with different diameters ranging from 50 nm to 2000 nm subjected to a constant biaxial compressive strain of 0.4% from the PMN-PT substrate. Details about the simulations are presented in the Supplementary Note 1. **b** The dependence of $\varepsilon_{top}/\varepsilon_{substrate}$ on the diameter of the nano-dot. $\varepsilon_{top}/\varepsilon_{substrate}$ represents the ratio of transferred strain on the central region of the top surface to the strain applied at the substrate. The data is well fitted by using an exponential equation $\varepsilon_{top}/\varepsilon_{substrate}$ = 1-exp(-2*d*). **c** Side view of the simulated ε distribution on a $d \sim 350$ nm nano-dot. We can find that, ε distribution shows a symmetric feature and the value of ε decreases along both the radius (r) and thickness (t) directions. The ε distribution can be further expressed as functions of r and t. For $t \leq 20$ nm, the ε distribution is homogeneous and the value of ε is approximately equal to that of the substrate (ε_{sub}). For $t \geq 20$ nm, the relationship between ε, t, and r can be expressed as

$$\varepsilon(t,r) = \varepsilon_{sub} \exp[-7.8 \times 10^{-3}(t-20)\exp(8.9 \times 10^{-3} r)], \quad (1)$$

where t ranges from 20 nm to 60 nm and r ranges from 0 to 175 nm. The color represents the value of ε at this region. The scale bar is 250 nm.

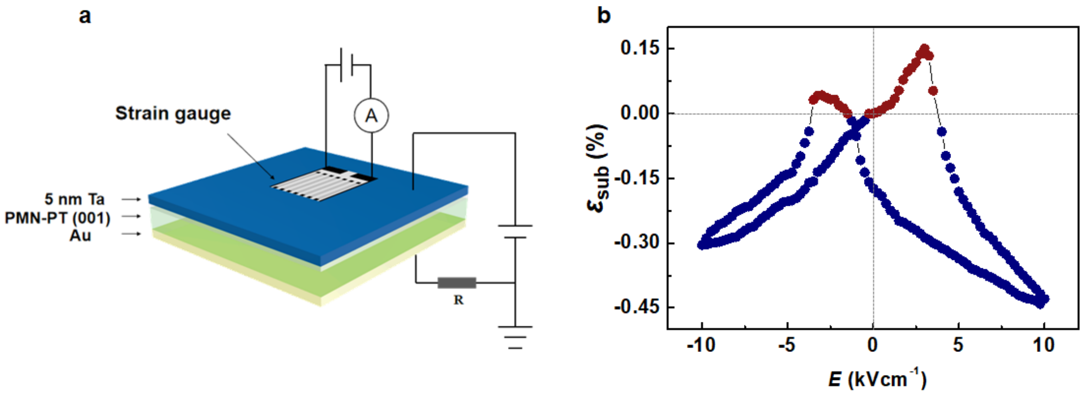

Supplementary Figure 4. a Schematic diagram for measuring the in-plane strain on the PMN-PT substrate. The external electric field was applied to the heterostructure through the Ta layer (blue layer) and Au electrode (yellow layer) by a voltage source meter (Keysight B2902A), which makes that an in-plane strain is generated at the PMN-PT substrate (green layer). To measure the in-plane transferred strain on the PMN-PT, a strain gauge is pasted to the top surface of the PMN-PT by using the strain glue (Kyowa CC-33A). The in-plane transferred strain (ε) can be calculated by using the formula $\varepsilon = \delta^*(\Delta R/R_0)$, where δ is the strain gauge sensitivity coefficient and here δ is fixed to be 2.0%; ΔR is the variation of the resistance with the change of in-plane transferred strain; R_0 is the initial resistance of the strain gauge and is equal to 120 Ω. **b** The measured E-ε_{sub} curve of the PMN-PT substrate. Based on simulated ε distribution shown in Supplementary Figure 3, ε_{ave} is calculated to be approximately 75% of the strain applied at the PMN-PT substrate (ε_{sub}) for the d ~ 350 nm $[Pt/Co/Ta]_{12}$ nano-dot and 91% for the d ~ 350 nm $[Pt/Co/Ta]_8$ nano-dot.

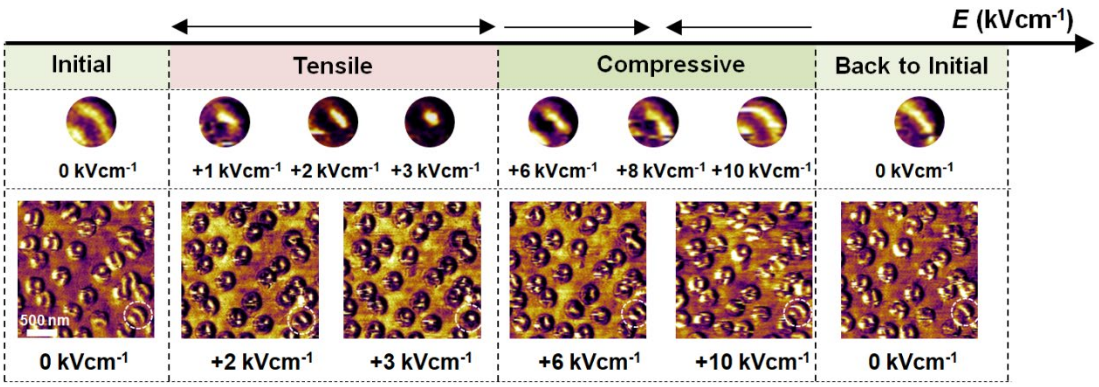

Supplementary Figure 5. The electric-field-induced magnetic domain evolution process in the $[Pt/Co/Ta]_{12}$ nano-dots with a diameter of 350 nm in an electric field ranging from +10 kV cm^{-1} to 0 kV cm^{-1}. The domain structures of the nano-dots on the upper row represent the detailed domain evolution process that was presented in the Fig. 2a in the main text. The MFM images below represent the domain evolution process in a much larger scale. The white dash circles mark the positions of the nano-dots on the upper row. The scale bar is 500 nm.

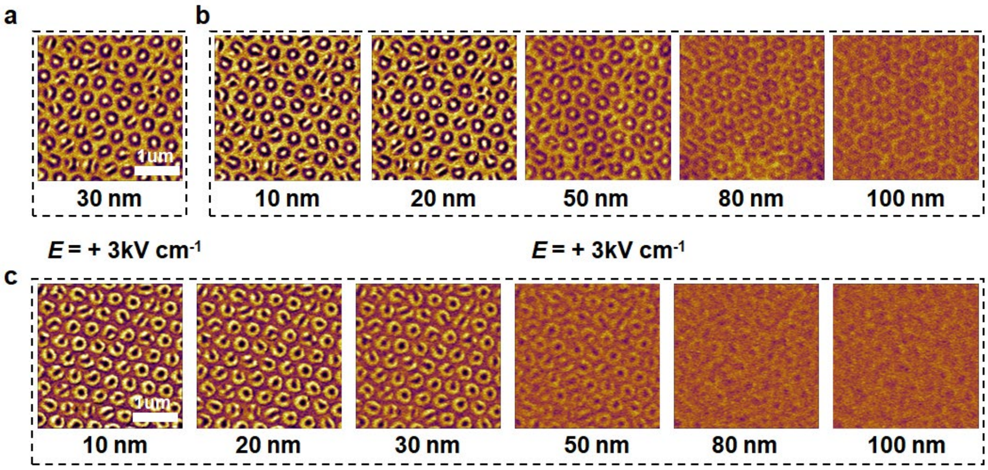

Supplementary Figure 6. a MFM image taken at E = +3 kV cm^{-1} under zero magnetic field for the d ~ 350 nm nano-dots of the [Pt/Co/Ta]$_{12}$ heterostructure. The distance between MFM tip and sample is fixed to be 30 nm. **b** MFM image taken at E = +3 kV cm^{-1} with different tip-sample distance. **c** MFM images taken at different tip-sample distance by reversing the direction of the magnetic field. The scale bar is 1 μm.

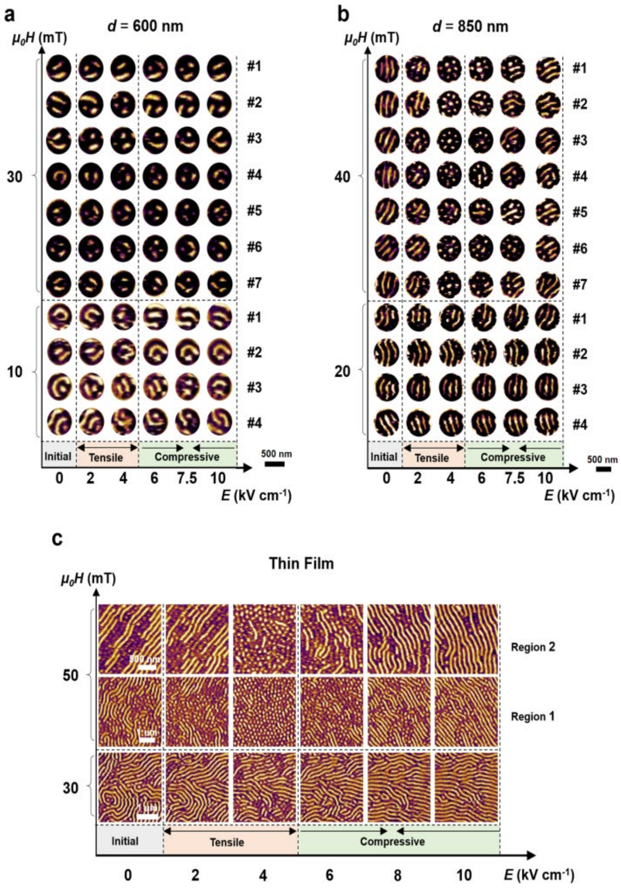

Supplementary Figure 7. Electric-field-induced binary switching of skyrmions in **a** 600 nm nano-dots, **b** 850 nm nano-dots and **c** thin film. For the d ~ 600 nm nano-dots, when a relative lower external magnetic field $\mu_0 H$ of 10 mT was applied, the stripe domain could not transform into skyrmions throughout E range. However, if the external magnetic field was increased to 30 mT, the skyrmion-stripe binary conversion was obtained. In the case of the 850 nm nano-dots and thin film, the critical magnetic field required for the stripe-skyrmion binary switching was established to be 40 mT and 50 mT, respectively. The scale bars of **a** and **b** are 1 μm. The scale bars of **c** are 500 nm and 1 μm.

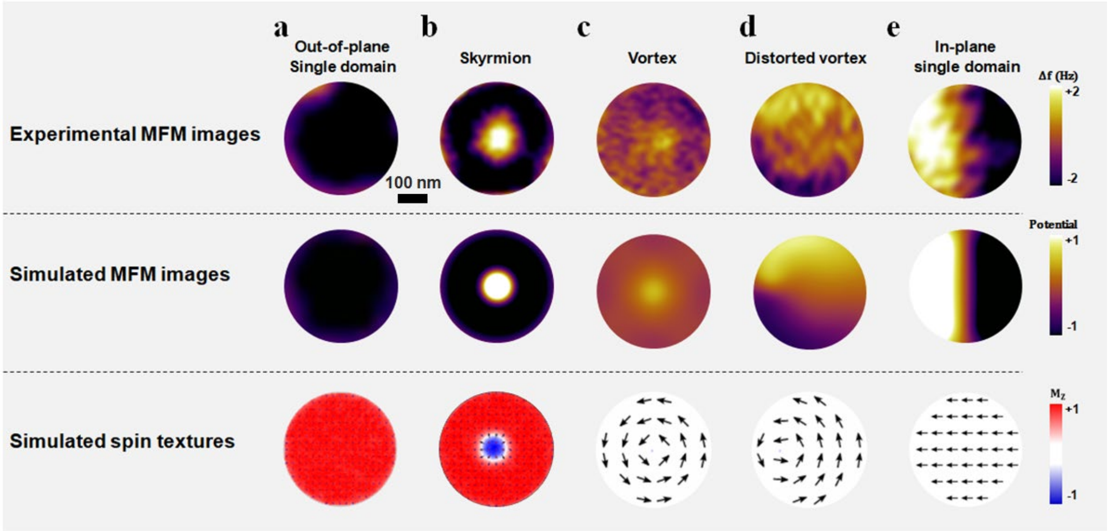

Supplementary Figure 8. Experimental MFM image (the top row), simulated MFM image (the middle row), and corresponding spin texture (the bottom row) for **a** an out-of-plane single domain, **b** skyrmion, **c** vortex, **d** distorted vortex (the core derivates from the centre position) and **e** in-plane single domain. The experimental MFM images shown in the top row represented the typical magnetic domain images observed in our experiments. First, we evaluated their possible spin textures by analyzing the contrast of the images and subsequently simulated the possible spin textures by using mumax3 software[1], as is shown in the bottom row. Then, we simulated the MFM images on the basis of the simulated spin textures by using a Green function method in MATLAB[2]; they were presented in the middle row. By comparing the simulated MFM images with the experimentally obtained images, we found that the simulated images were highly consistent with the experimental ones, suggesting that the actual spin textures of magnetic domains observed in our experiments were quite similar to the simulated textures. The scale bar is 100 nm.

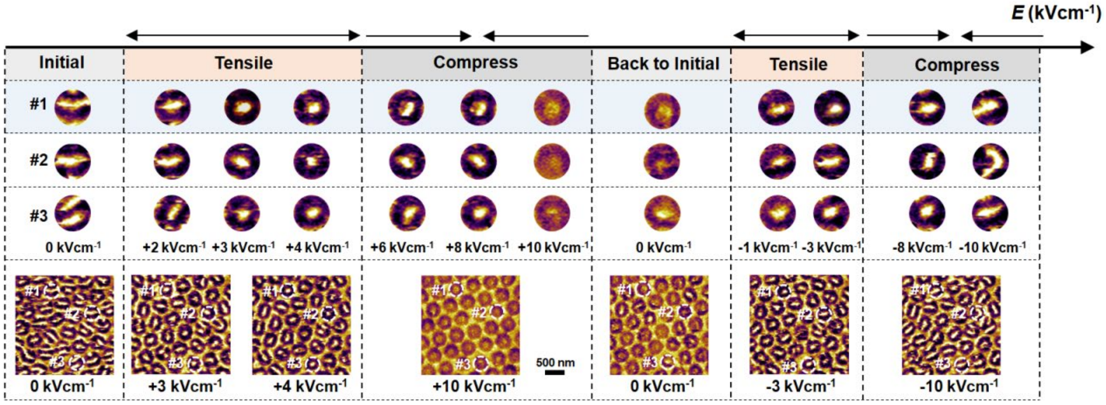

Supplementary Figure 9. The detailed process of the electric-field-induced magnetic domain evolution of three typical d ~ 350 nm $[Pt/Co/Ta]_8$ nano-dots (#1, #2, and #3) in an electric field ranging from +10 kV cm^{-1} to -10 kV cm^{-1}. The #1 nano-dots are the same with that of Fig.2b in the main text and the corresponding MFM images (blue background) demonstrate the detailed magnetic domain evolution process. The MFM images below represent the domain evolution process in a much larger scale. The white dash circles mark the positions of the nano-dots on the upper row. The scale bar is 500 nm.

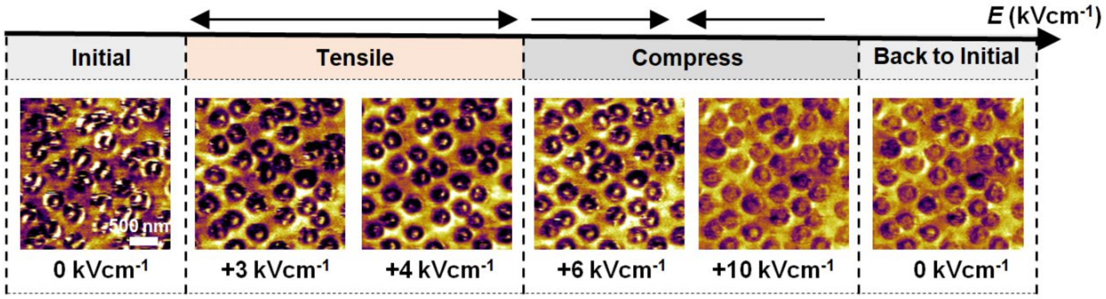

Supplementary Figure 10. The repetition of the electric-field-induced magnetic domain evolution in another $[Pt/Co/Ta]_8$ heterostructure. A stripe-skyrmion-vortex multi-state conversation could be observed in the positive numerical range from 0 kV cm^{-1} to +10 kV cm^{-1}. After *E* was removed, the vortex could be retained. This tendency was similar to that observed in the sample shown in Fig. 2b in the main text. The scale bar is 500 nm.

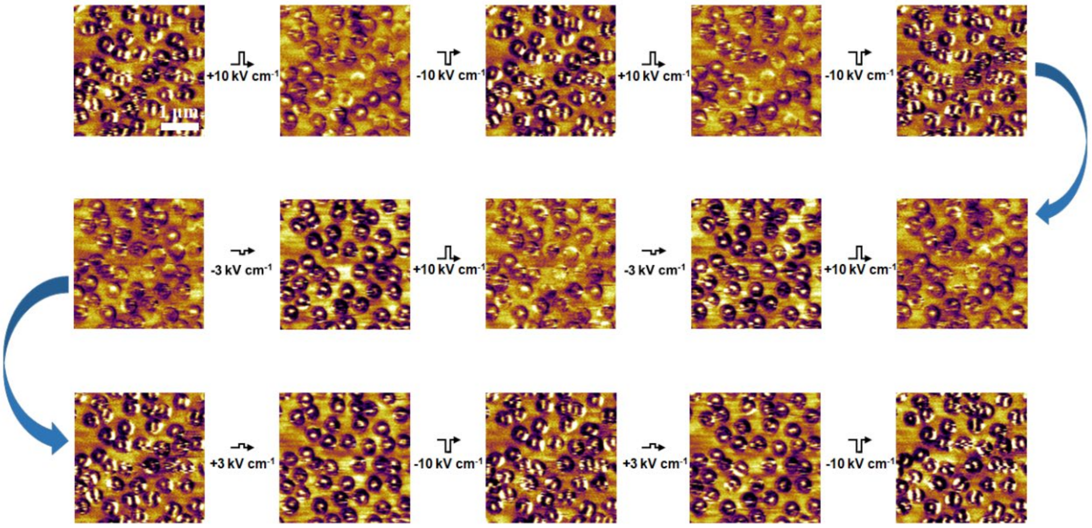

Supplementary Figure 11. The MFM images of the three-states switching induced by using 1 ms pulses of E = ±3 kV cm^{-1}, +10 kV cm^{-1}, and -10 kV cm^{-1}, which corresponds to the generation of skyrmions, vortex, and stripe, respectively. The scale bar is 1 μm.

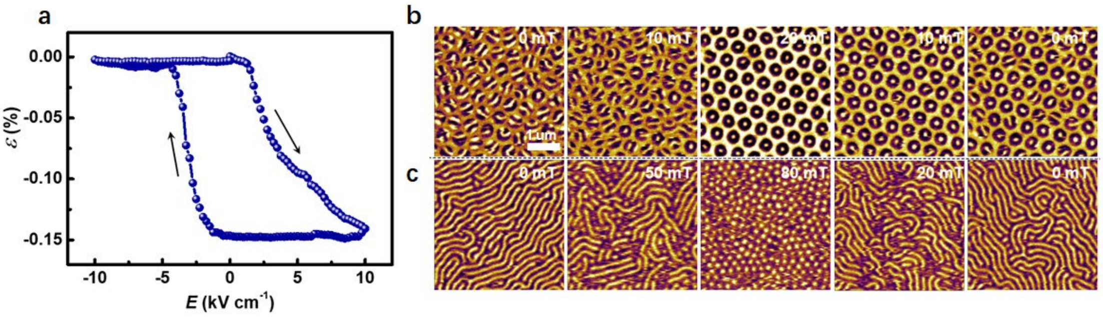

Supplementary Figure 12. a The remanent strain of $[Pt/Co/Ta]_8$ heterostructure for different electric field (*E*). The remanent strain is measured after decreasing *E* from a target value to zero. **b** Magnetic field-dependent domain evolution process of the $[Pt/Co/Ta]_{12}$ heterostructure. **c** Magnetic field-dependent domain evolution process of the $[Pt/Co/Ta]_{12}$ continuous thin film. The scale bar is 1 μm.

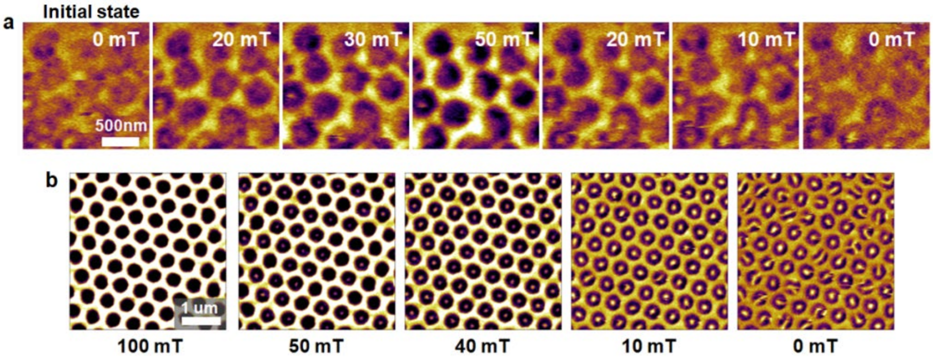

Supplementary Figure 13. a Magnetic field-dependent domain evolution process of the $[Pt/Co/Ta]_8$ heterostructure. These images are taken at $E = 0$ kV cm^{-1} after decreasing E from +10 kV cm^{-1}. **b** Magnetic field-dependent domain evolution process with the magnetic field decreasing from 100 mT to 0 mT. The scale bar of **a** and **b** is 500 nm and 1 μm, respectively.

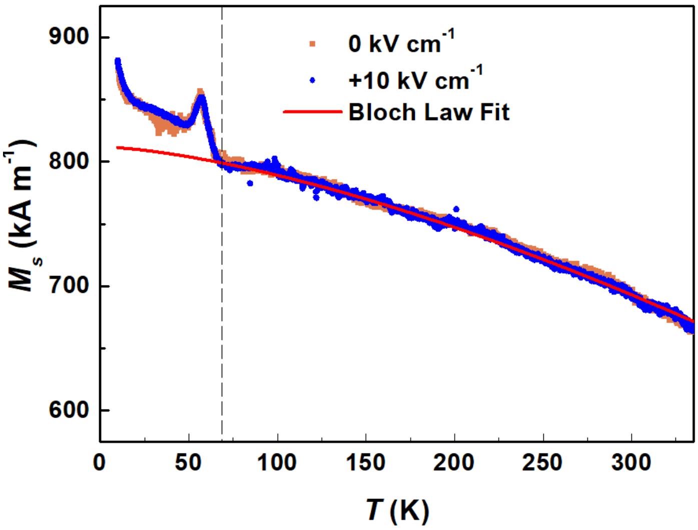

Supplementary Figure 14. Temperature-dependent saturation magnetization M_s(T) of the $[Pt/Co/Ta]_{12}$ continuous thin film at E = 0 kV cm^{-1} (orange dots) and +10 kV cm^{-1} (blue dots). The experimental dots are fitted by using the Bloch law (red line).

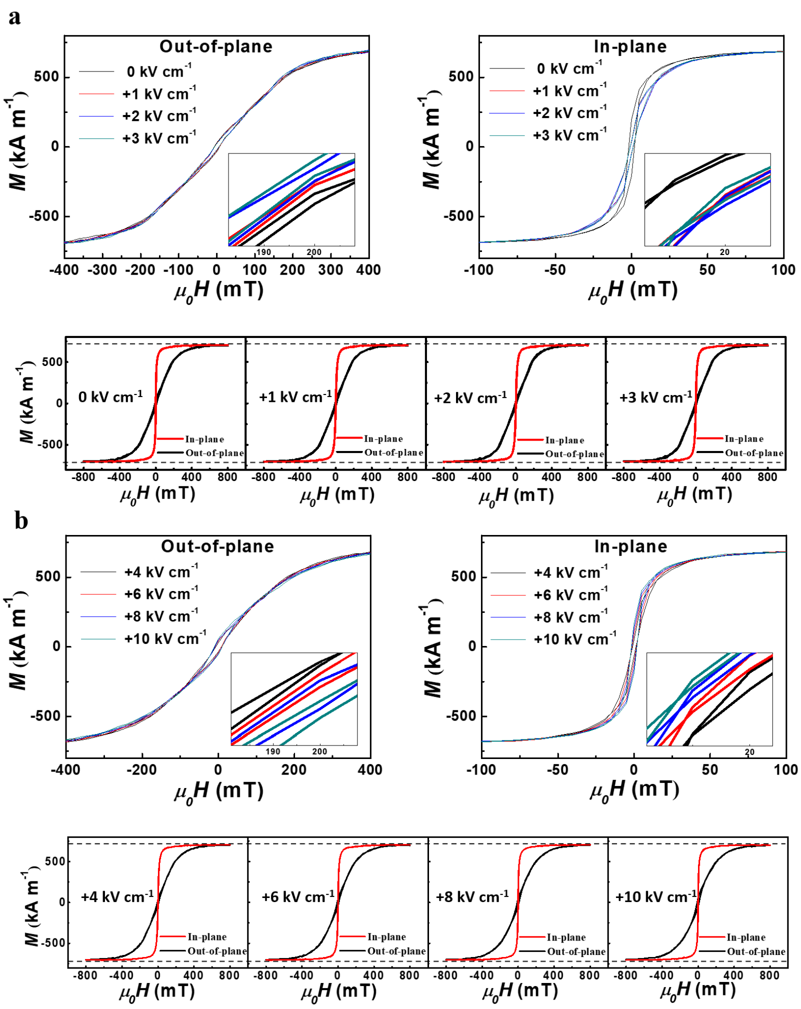

Supplementary Figure 15. Out-of-plane (right) and in-plane (left) hysteresis loops of the $d \sim 350$ nm $[Pt/Co/Ta]_{12}$ nano-dot at E ranging **a** from 0 kV cm^{-1} to +3 kV cm^{-1} (the corresponding strain exhibited a tensile type) and **c** from +4 kV cm^{-1} to +10 kV cm^{-1} (the corresponding strain exhibited a compressive type). The hysteresis loops were measured with the application of an external E by using a physical property measurement system (PPMS, Quantum Design). To show the variation of hysteresis loops with the change of E more clearly, the amplification of the hysteresis loops is presented in the insets. The electric-field-induced variation of magnetic anisotropy could be quantitatively evaluated by the value of effective magnetic anisotropy constant (K_{eff}), which was calculated by using the area difference between the in-plane and out-of-plane magnetization curves. The positive and negative K_{eff} represent the perpendicular magnetic anisotropy (PMA) and in-plane magnetic anisotropy (IMA), respectively.

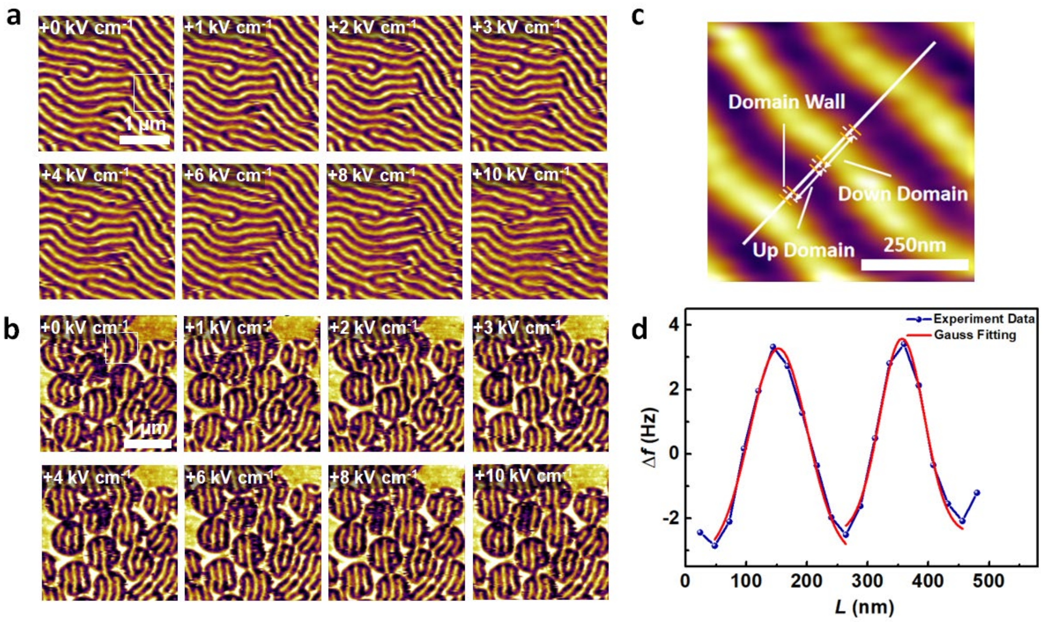

Supplementary Figure 16. The steps for establishing the values of *w*. (i) First, high-quality *E*-dependent MFM images of the $[Pt/Co/Ta]_{12}$ continuous thin film and *d* ~ 850 nm nano-dots were obtained without external magnetic field, as shown in **a** and **b**. **c** is an amplification MFM image of the region enclosed by the white box in **a**. In this image, the regions that represent the domain wall, up domain and down domain are clearly marked. **c** is the typical contrast variation extracted from the MFM image. The distance (*L*) between two neighboring contrast peaks is proposed to be a domain period (*w*). The experimental data is fitted by Gauss function (red line) to establish the position of the peak. The scale bar is 1 μm.

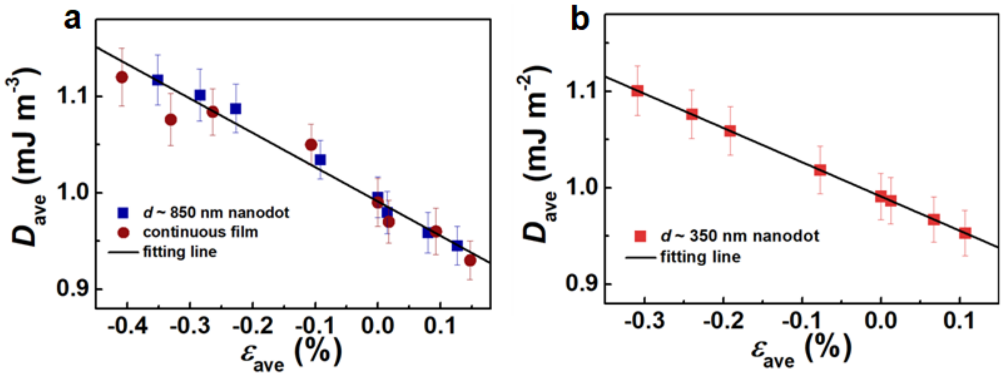

Supplementary Figure 17. a Dependence of the experimentally established D_{ave} on ε_{ave} for the continuous thin film (red circle) and d ~ 850 nm nano-dot (blue square). The black line represents the fitted equation for the data points of both continuous thin film and d ~ 850 nm nano-dot. **b** Dependence of the evaluated D_{ave} (red square) on ε_{ave} for d ~ 350 nm nano-dot. The black line represents the fitted equation for the data. The error margin of D_{ave} at different E is added based on that of σ_{DW}, A, M_s, and K_{ave}.

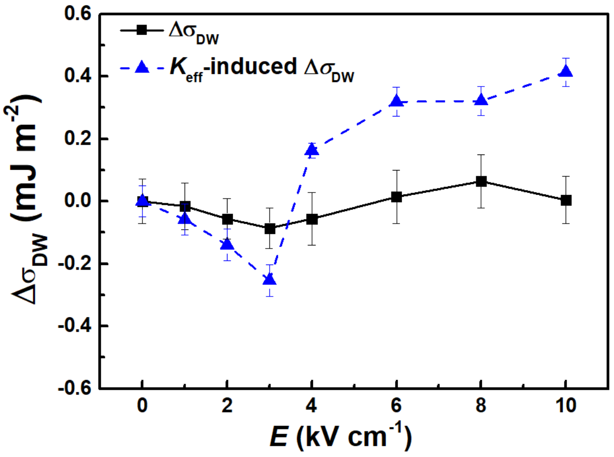

Supplementary Figure 18. E-dependent $\Delta\sigma_{DW}$ of the continuous thin film. $\Delta\sigma_{DW}$ represents the variation of σ_{DW} and is equal to $\sigma_{DW}(E)$-$\sigma_{DW}(0)$. Black dotted line represents E-dependent $\Delta\sigma_{DW}$ that is derived directly based on the E-dependent w presented in "#1" of Supplementary Figure 16. The blue dotted line represents the $\Delta\sigma_{DW}$ that is derived based on σ_{DW} calculated by using K and D. To demonstrate the influence of K_{eff} on $\Delta\sigma_{DW}$, the value of D is fixed to be D_0 that is equal to the value of D on the continuous thin film at $E = 0$ kV/cm. Since E-dependent K_{eff} is well established in our experiments, the values of σ_{DW} at different K_{eff} can be calculated. $\Delta\sigma_{DW}$ is equal to the difference between $\sigma_{DW}(K)$ and $\sigma_{DW}(K_0)$, namely, $\Delta\sigma_{DW} = \sigma_{DW}(K) - \sigma_{DW}(K_0)$. The error margin of K_{ave} at different E is added by measuring different regions of the continuous thin film.

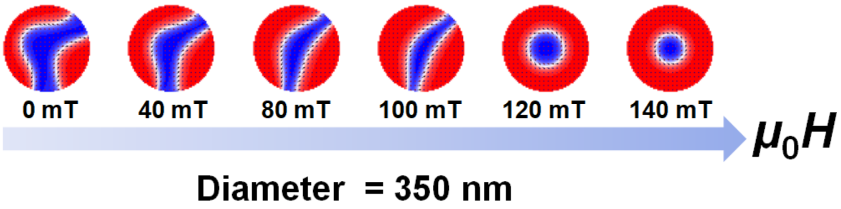

Supplementary Figure 19. Micromagnetic simulation of the creation of skyrmions from stripe domain walls in a $d \sim 350$ nm nano-dot on the basis of the magnetic parameters of the $[Pt/Co/Ta]_{12}$ heterostructure. As the out-of-plane magnetic field increased from $\mu_0 H$ = 0 mT to 140 mT, the stripe domain gradually transformed into a single skyrmion.

s_{11}^E(m^2 N^{-1})	s_{33}^E(m^2 N^{-1})	s_{12}^E(m^2 N^{-1})	s_{13}^E(m^2 N^{-1})	s_{44}^E(m^2 N^{-1})
69.0	119.6	-11.1	-55.7	14.5
s_{66}^E(m^2 N^{-1})	d_{33}^E(CN^{-1})	d_{31}^E(CN^{-1})	$\varepsilon_{33}/\varepsilon_0$	
15.2	-1338	2820	6650	

Supplementary Table 1. The elastic and piezoelectric parameters of PMN-PT[3]. ε_0 denotes the dielectric constant of air.

			thin film				$d \sim 850$ nm nano-dot			
E (kV cm^{-1})	M_s (kA m^{-1})	A (pJ m^{-1})	ω (nm)	σ_{DW} (mJ m^{-2})	K_{ave} (×10^{5}J m^{-3})	D_{ave} (mJ m^{-2})	ω (nm)	σ_{DW} (mJ m^{-2})	K_{ave} (×10^{5}J m^{-3})	D_{ave} (mJ m^{-2})
0	697±7	16.9±0.2	193±3	2.35±0.08	-1.10±0.02	0.987±0.027	171±3	2.02±0.08	-0.98±0.01	0.995±0.021
1	697±7	16.9±0.2	192±3	2.35±0.08	-1.08±0.02	0.971±0.024	171±2	2.03±0.07	-0.96±0.01	0.980±0.022
2	697±7	16.9±0.2	190±3	2.30±0.07	-1.04±0.02	0.959±0.028	172±3	2.04±0.08	-0.93±0.02	0.955±0.026
3	697±7	16.9±0.2	188±2	2.27±0.07	-1.00±0.02	0.933±0.022	171±3	2.02±0.08	-0.91±0.01	0.942±0.020
4	697±7	16.9±0.2	190±4	2.30±0.09	-1.17±0.01	1.054±0.023	168±2	1.98±0.06	-1.01±0.02	1.034±0.020
6	697±7	16.9±0.2	194±4	2.37±0.09	-1.23±0.02	1.084±0.026	167±4	1.96±0.09	-1.07±0.02	1.087±0.025
8	697±7	16.9±0.2	197±5	2.42±0.09	-1.24±0.02	1.076±0.029	170±3	2.00±0.08	-1.10±0.01	1.102±0.027
10	697±7	16.9±0.2	193±3	2.36±0.08	-1.27±0.02	1.118±0.024	173±4	2.05±0.09	-1.15±0.02	1.116±0.026

Supplementary Table 2. The experimentally derived w, M_s, K_{ave}, A, σ_{DW} and D_{ave} for continuous thin film and $d \sim 850$ nm nano-dot.

Supplementary Note 1. The strain distribution is simulated by using the Abaqus v6.14-1 software. The material parameters of PMN-PT used for the simulation are given in Supplementary Table 1. The elastic moduli and Poisson's constants of Ta, Co and Pt are given as 186 GPa, 215 GPa, 171 GPa, and 0.34, 0.33, 0.34, respectively. Here, the multi-layered nano-dot is simplified into a homogeneous structure, where the elastic parameters are approximately calculated as 190 GPa and 0.34.

Supplementary Note 2. The M_s(T) curve can be fitted with the Bloch law[4,5]

$$M_s(\mathrm{T}) = M_0(1 - b/T^{3/2}) \qquad (2)$$

,where M_0 is the saturation magnetization at zero Kelvin and b is a constant. The constant b can be further given by

$$A = \frac{nk_B S^2}{a}(\frac{C}{b})^{2/3} \qquad (3)$$

,where n = 4 is the coordination number for a fcc lattice[4,5] (the Co that deposited at room-temperature using sputter generally crystallizes into the fcc structure[5,6]), k_B is the Boltzmann constant that takes a value of 1.380649×10^{-23} J K^{-1}, S is the spin per atom that takes the value of 1[5,7], C is a constant that takes the value 0.0294 for an fcc lattice[4,5], and a is the lattice constant of fcc Co that takes a value of 0.3536 nm[5,8]. Thus, if the value of b is established, we can directly calculate the value of A by using Supplementary Equation 2. To determine the value of b, we have measured the temperature-dependent saturation magnetization of the $[\mathrm{Pt/Co/Ta}]_{12}$ heterostructure at zero electrical field, as shown in Supplementary Figure 14. We find that the M_s(T) curve agree well with the Bloch law over the temperature range of 60 K - 330 K. However, when the temperature decreases below 60 K, the curve deviates from the Bloch law. Such deviations may be attributed to the pronounced role of the Ruderman-Kittel-Kasuya-Yosida (RKKY) interaction at the low-temperature range (below 60 K)[9,10]. The corresponding value of A can be calculated to be 16.9±0.2 pJ m^{-1}. The error bar is added by fitting the M_s(T) cure within different temperature range and such a value of A falls within the reported range of A for the Co-based thin film[7,10,11].

To investigate the effect of strain on the variation of A, we have measured the temperature-dependent saturation magnetization of the $[\mathrm{Pt/Co/Ta}]_{12}$ continuous thin film at +10 kV cm^{-1} where the PMN-PT substrate exhibits the maximum strain. As shown in Supplementary Figure 14, the M_s(T) curve keeps nearly unchanged at the whole temperature range of 10 - 330 K compared with that measured at a zero electrical field. Thus, the value of A should keep nearly unchanged under such a strain. That is to say, the value of A is insensitive to the strain in our experiments. On the other hand, previous experimental reports demonstrated that when a strain of 0.3% was applied on a FeGe thin plate, the value of T_c just varied by 3.3 K[12]. Such a slight variation of T_c only corresponds to a few percentage variation of A compared with that at the ambient pressure (T_c = 280 K)[12]. For the $[\mathrm{Pt/Co/Ta}]_{12}$ heterostructure in our experiments, the maximum tensile strain is 0.026% and the maximum compressive stain is 0.07%. These values are nearly one order of magnitude lower than that applied on the FeGe. Thus, we believe that the value of A is slightly affected by such a strain and it is reasonable for us to treat A as a strain-independent parameter in our experiments.

Supplementary Note 3. In Supplementary Figure 17a, we show a series of D_{ave} derived at different ε_{ave} on the continuous thin film. We can find that the absolute value of D_{ave} decreases

with increasing tensile strain ($\varepsilon_{ave}<0$) while increases with increasing compressive strain ($\varepsilon_{ave}>0$). Such a change tendency of D_{ave} with ε_{ave} is consistent with that reported in recent literatures[12-16], which thus validates our results. We have also derived the ε_{ave}-dependent D_{ave} on the d ~ 850 nm nano-dot, as shown in Supplementary Figure 17a. The corresponding change tendency of D_{ave} with ε_{ave} is similar to that on the continuous thin film, which confirms that D_{ave} is closely coupled with ε_{ave}. Moreover, we find that the ε_{ave}-dependent D_{ave} on the d ~ 850 nm nano-dot nearly overlaps that on the continuous thin film though the ε_{ave} range of the continuous thin film is smaller than that of the d ~ 850 nm nano-dot. This feature suggests that the relationship between D_{ave} and ε_{ave} is little affected by the geometrically confined effect, namely, the relationship between D_{ave} and ε_{ave} is approximately fixed no matter on the continuous thin film or the nano-dots with different diameters. That can be attributed to the intrinsic feature of D. As known to us, D directly originates from the spin-orbital coupling effect of the film interface. Since the spin-orbital coupling effect depends on the electronic structure of the film, it can be little affected by the geometrical confinement. However, the strain may show a significant influence on D because it can affect the electronic structure and spin-orbital coupling effect of the film by tuning its lattice parameter[13,17,18]. Thus, we have observed that the value of D can be changed by strain and the relationship between D and strain is little affected by the geometrical confinement. To evaluate ε_{ave}-dependent D_{ave} on the d ~ 350 nm nano-dot, the relationship between D_{ave} and ε_{ave} should be established. In recent reports[15,16], D_{ave} is proposed to vary with ε_{ave} approximately in a linear manner. In our work, a linear equation can also be derived by fitting the ε_{ave}-dependent D_{ave} for both the continuous thin film and d ~ 850 nm nano-dot (see Supplementary Figure 17a). Based on the fitted ε_{ave}-D_{ave} equation and the ε_{ave} on the d ~ 350 nm nano-dot, we can hence derive the values of D_{ave} on the d ~ 350 nm nano-dot at different ε_{ave}, as shown in Supplementary Figure 17b. The error bar is added by fitting different ε_{ave}-D_{ave} equations.

Supplementary Note 4. As shown in Supplementary Table 2, the value of w for the continuous thin film shows a slight change with E. Based on the E-dependent w, we have further calculated the corresponding domain wall surface energy density (σ_{DW}). Since σ_{DW} is positively correlated with w, the value of σ_{DW} also varies slightly with that of E. As known to us, σ_{DW} is directly coupled with D and K_{eff}. In our experiments, we have confirmed that the value of K_{eff} varies significantly with that of E on the continuous thin film. If D is assumed to be fixed, the variation K_{eff} should lead to a significant change of σ_{DW} (see Supplementary Figure 18). Details about establishing the change of σ_{DW} induced by the variation K_{eff} are presented in the caption of Supplementary Figure 18. Since σ_{DW} is positively correlated with w, a significant change of w for the stripe domains should also be observed in the MFM images, as is the case reported by Dai *et al*[19]. However, our results demonstrate that w only changes slightly with E, as shown in Supplementary Figures 16a and b. Such a discrepancy suggests that D should also be changed by E (or ε) to offset the influence from the variation of K_{eff} on w. Therefore, we propose that both K_{eff} and D are changed by E (or ε) on the continuous thin film and an interplay of the two parameters results in a delicate balance of the stripe domain.

Supplementary Note 5. Based on the simulated results (see Supplementary Figure 3), the ε distribution on the d ~ 350 nm nano-dot can be expressed as

$$\varepsilon(t,r) = \begin{cases} 1.3\varepsilon_{\text{ave}} & 0\text{nm} \le t \le 20\text{nm} \\ 1.3\varepsilon_{\text{ave}} \exp[-7.8\times 10^{-3}(t-20)\exp(8.9\times 10^{-3}r)] & 20\text{nm} < t \le 60\text{nm} \end{cases} \quad (4)$$

,where ε_{ave} is the average strain of the nano-dot, t is the thickness of the nano-dot (ranging from 0 to 60 nm), and r is the radius of the nano-dot (ranging from 0 to 175 nm). On the other hand, by fitting ε_{ave}-K_{eff} and ε_{ave}-D_{ave} curves in Figs. 4a and 4b in the main text, the ε_{ave}-dependent K_{ave} and D_{ave} can be given as

$$K_{\text{ave}} = 5.1\times 10^{6}\varepsilon_{\text{ave}} + K_0 \quad (5)$$

$$D_{\text{ave}} = -3.6\times 10\varepsilon_{\text{ave}} + D_0 \quad (6)$$

,where K_0 or D_0 is effective magnetic anisotropy/DMI constant at the unstrained state and K_{ave} or D_{ave} is the average K_{eff} or D of the nano-dot. By combining the relationship between ε and K_{ave} and D_{ave} with Supplementary Equation 4, the K_{eff} and D distributions on the nano-dot can be expressed as

$$K_{\text{eff}}(\text{t},\text{r}) = \begin{cases} 1.3(K_{\text{ave}}-K_0)+K_0 & 0\text{nm} \le t \le 20\text{nm} \\ 1.3(K_{\text{ave}}-K_0)\exp[-7.8\times 10^{-3}(t-20)\exp(8.9\times 10^{-3}r)]+K_0 & 20\text{nm} < t \le 60\text{nm} \end{cases} \quad (7)$$

$$D(\text{t},\text{r}) = \begin{cases} 1.3(D_{\text{ave}}-D_0)+D_0 & 0\text{nm} \le t \le 20\text{nm} \\ 1.3(D_{\text{ave}}-D_0)\exp[-7.8\times 10^{-3}(t-20)\exp(8.9\times 10^{-3}r)]+D_0 & 20\text{nm} < t \le 60\text{nm} \end{cases}$$

(8)

,where K_0 and D_0 are effective magnetic anisotropy and DMI constant at the unstrained state.

To reduce the computation load of the simulations, we have simplified the model. Along the thickness direction, the model is divided into three layers (each layer is 20 nm in thickness). For the bottom layer ($t \le 20$ nm), since the K_{eff} and D distributions are homogeneous, it is no need to be simplified. In the case of the middle layer (20 nm < $t \le 40$ nm) and top layer (40 nm < $t \le 60$ nm), we propose that the K and D distributions along the thickness are homogenous for a fixed r but varies linearly with r. Then, the Supplementary Equations 5 and 6 can be simplified as:

$$K_{\text{eff}}(\text{t},\text{r}) = \begin{cases} 1.3(K_{\text{ave}}-K_0)+K_0 & 0\text{nm} \le t \le 20\text{nm} \\ 1.3(K_{\text{ave}}-K_0)(0.9-4.6\times 10^{-4}r)+K_0 & 20\text{nm} < t \le 40\text{nm} \\ 1.3(K_{\text{ave}}-K_0)(0.6-1.4\times 10^{-3}r)+K_0 & 40\text{nm} < t \le 60\text{nm} \end{cases} \quad (9)$$

$$D(\text{t},\text{r}) = \begin{cases} 1.3(D_{\text{ave}}-D_0)+D_0 & 0\text{nm} \le t \le 20\text{nm} \\ 1.3(D_{\text{ave}}-D_0)(0.9-4.6\times 10^{-4}r)+D_0 & 20\text{nm} < t \le 40\text{nm} \\ 1.3(D_{\text{ave}}-D_0)(0.6-1.4\times 10^{-3}r)+D_0 & 40\text{nm} < t \le 60\text{nm} \end{cases} \quad (10)$$

The dynamics of magnetization are governed by the Landau-Lifshitz-Gilbert (LLG) equation[20,21]

$$\frac{\mathrm{d}\boldsymbol{m}}{\mathrm{d}t} = -\gamma_0 \mathbf{m}\times\mathbf{h}_{\text{eff}} + \alpha\left(\mathbf{m}\times\frac{\mathrm{d}\boldsymbol{m}}{\mathrm{d}t}\right), \quad (11)$$

where t is the time, γ_0 is the gyromagnetic ratio with absolute value, and α is the Gilbert damping coefficient. In addition, $\boldsymbol{m} = \mathbf{M}/M_s$ is the reduced magnetization with saturation magnetization M_s, $\mathbf{h}_{\text{eff}} = -\partial E_{\text{den}}/\partial\mathbf{m}$ is the effective field, and the energy density E_{den} as a function of $\boldsymbol{m}$ includes the exchange energy, anisotropy energy, demagnetization energy, and Dzyaloshinskii-Moriya energy. In the micromagnetic simulations, the following magnetic parameters are used: M_s= 6.9 × 10^5 A m^{-1}, A = 1.7×10^{-11} J m^{-1}, D = 0.10 ~ 2.50 mJ m^{-2}, K_{eff} = 0.00 ~ -2.50 × 10^5 J m^{-3}. Furthermore, the Gilbert damping coefficient is 0.3. The simulated nano-dot has a diameter of 350 nm and thickness of 60 nm, and the mesh size is 5 × 5 × 20 nm^{3}[22]. The micromagnetic simulations are performed with the software package OOMMF. Notable, the simulation is performed at zero temperature and no thermal field is introduced.

Supplementary References

1. Vansteenkiste, A. et al. The design and verification of MuMax3. *AIP Adv.* **107**, 0-22 (2014).
2. Saito, H., Chen, J. & Ishio, S. Description of magnetic force microscopy by three-dimensional

tip Green's function for sample magnetic charges. Journal of magnetism and magnetic materials. *J. Mag. Mag. Mater.* **191**, 153-161 (1999).
3. Zhang, R., Jiang, B. & Cao W. Elastic, piezoelectric, and dielectric properties of multidomain $0.67Pb(Mg_{1/3}Nb_{2/3})O_3$-$0.33PbTiO_3$ single crystals. *J.Appl. Phys*. **90**, 3471-3475 (2001).
4. Chikazumi, S. *Physics of Ferromagnetism,* 2nd ed. (Oxford University Press, Oxford, 1997).
5. Shahbazi, K. et al. Domain-wall motion and interfacial Dzyaloshinskii-Moriya interactions in Pt/Co/Ir(t_{Ir})/Ta multilayers. *Phys. Rev. B* **99**, 094409 (2019).
6. Wells, A. W., Shepley, P. M., Marrows, C. H. & Moore, T. A. Effect of interfacial intermixing on the Dzyaloshinskii-Moriya interaction in Pt/Co/Pt. *Phys. Rev. B* **95**, 054428 (2017).
7. Balashov, T. et al. Magnetic anisotropy and magnetization dynamics of individual atoms and clusters of Fe and Co on Pt (111). *Phys. Rev. Lett*. **102**, 257203 (2009).
8. Liu, X. et al. Exchange stiffness, magnetization, and spin waves in cubic and hexagonal phases of cobalt. *Phys. Rev. B* **53**, 12166 (1996).
9. Tahiri, N., Ez-Zahraouy, H. & Benyoussef, A. Multilayer transition in a spin 3/2 Blume–Capel model with RKKY interaction. *Physica A* **388**, 3426-3432 (2009).
10. Knepper, J. W. & Yang, F. Y. Oscillatory interlayer coupling in Co/Pt multilayers with perpendicular anisotropy. *Phy. Rev. B* **71**, 224403 (2005).
11. Talapatra, A. & Mohanty, J. Laser induced local modification of magnetic domain in Co/Pt multilayer. *J. Magn. Magn. Mater.* **418**, 224-230 (2016).
12. Shibata, K. et al. Large anisotropic deformation of skyrmions in strained crystal. *Nature Nanotech*. **10**, 589-592 (2015).
13. Koretsune, T., Nagaosa, N. & Arita, R. Control of Dzyaloshinskii-Moriya interaction in $Mn_{1-x}Fe_xGe$: a first-principles study. *Sci. Rep.* **5**, 13302 (2015).
14. Kitchaev, D. A., Beyerlein, I. J. & Van der Ven, A. Phenomenology of chiral Dzyaloshinskii-Moriya interactions in strained materials. *Phys. Rev. B* **98**, 214414 (2018).
15. Zhang, W. et al. Enhancement of Interfacial Dzyaloshinskii-Moriya Interaction: A Comprehensive Investigation of Magnetic Dynamics. *Physical Review Applied*, **12**, 064031 (2019).
16. Gusev, N. S., Sadovnikov, A. V., Nikitov, S. A., Sapozhnikov, M. V. & Udalov, O. G. Manipulation of the Dzyaloshinskii–Moriya Interaction in Co/Pt Multilayers with Strain. *Phys. Rev. Lett*. **124**, 157202 (2020).
17. Kautzsch, L., Bocarsly, J. D., Felser, C., Wilson, S. D. & Seshadri, R. Controlling Dzyaloshinskii-Moriya interactions in the skyrmion host candidates $FePd_{1-x}Pt_xMo_3N$. *Phys. Rev. Materials* **4**, 024412 (2020).
18. Grytsiuk, S., et al. Ab initio analysis of magnetic properties of the prototype B20 chiral magnet FeGe. *Phys. Rev. B* **100**, 214406 (2019).
19. Dai, G., Xing, X., Shen, Y. & Deng, X. Stress tunable magnetic stripe domains in flexible $Fe_{81}Ga_{19}$ films. *J. Phys. D: Appl. Phys.* **53**, 055001 (2020).
20. Gilbert, T. L. A Lagrangian formulation of the gyromagnetic equation of the magnetization field. *Phys. Rev.* **100**, 1243 (1955).
21. Landau, L. & Lifshitz, E. On the Theory of the Dispersion of Magnetic Permeability in Ferromagnetic Bodies. *Phys. Z. Sowjetunion* **8**, 153-164 (1935).
22. Donahue, M. J. & Porter, D. G. OOMMF User's Guide, Version 1.0 Interagency Report NISTIR 6376 (National Institute of Standards and Technology, Gaithersburg, MD, 1999).
